# Perfect spin filtering effect and negative differential behavior in phosphorus-doped zigzag graphene nanoribbons

**DOI:** 10.1038/srep15966

**Published:** 2015-10-30

**Authors:** Fei Zou, Lin Zhu, Kailun Yao

**Affiliations:** 1 School of Physics and Wuhan National High Magnetic Field Center, Huazhong University of Science and Technology, Wuhan 430074, China; 2 International Center of Materials Physics, Chinese Academy of Science, Shenyang 110015, China

## Abstract

On the basis of the density functional theory combined with the Keldysh nonequilibrium Green’s function method, we investigate the spin-dependent transport properties of single-edge phosphorus-doped ZGNR systems with different widths. The results show a perfect spin filtering effect reaching 100% at a wide bias range in both parallel (P) and antiparallel (AP) spin configurations for all systems, especially for 6-ZGNR-P system. Instructively, for the AP spin configuration, the spin down current of the 4-ZGNR-P system exhibits a negative differential effect. By analyzing the transmission spectrum and the spin-resolved band structures of the electrodes, we elucidate the mechanism for these peculiar properties. Our findings provide a new way to produce multifunctional spintronic devices based on phosphorus-doped zigzag graphene nanoribbons.

Graphene has attracted tremendous attention due to its exceptional properties, including high carrier mobility and long spin relaxation time etc^1^,^2^,^3^. The one-dimensional graphene nanoribbons (GNRs) with confined widths and atomically smooth edges can be prepared by cutting graphene^4^,^5^,^6^,^7^. The properties of GNRs depend on its edge shapes, and many investigations have focused on the zigzag-edged graphene nanoribbons (ZGNRs) due to the existence of edge states, which result in the spin polarization in ZGNRs. That is to say, the magnetisms concentrated on the edge atoms^8^,^9^,^10^,^11^. The edge states cause the spin polarization and the local magnetism, which can be understood from the Stoner magnetism of *sp* electrons^12^,^13^. Therefore, the magnetization of ZGNRs can be controlled by using external magnetic fields or through chemistry methods, making it show many peculiar properties. Now, ZGNRs electronics have ignited intense research due to its fascinating physical properties, such as spin filtering^14^,^15^,^16^,^17^, negative differential resistance^18^, etc, that have potential applications in spintronics.

Substitutional doping in ZGNRs draws many scientists’ attention. Many studies have shown that edged doping is more stable, and edge impurities can suppress the spin polarization of the doped edge for single atom substitution^19^,^20^,^21^,^22^,^23^,^24^. Then a single atom doping on the ZGNR edges can manipulate the spin transport properties^20^,^24^,^25^,^26^,^27^. In addition, the width of the ZGNRs is also crucial for electronic scattering, generally, different widths represent various unique properties^25^,^28^. Cruz-Silva *et al.*^20^ show that the edge or center phosphorus-doped ZGNRs exhibit both donor and acceptor states. Zhou *et al.*^29^,^30^ show that rectification and negative differential resistance behavior exist in their center phosphorus-doped armchair GNRs (AGNRs) devices, and it can be modulated by the width of AGNRs.

Up to now, there are few reports about the spin transport properties of edged phosphorus-doped ZGNRs. Here, we carry out first-principles transport calculations to study the edge phosphorus-doped ZGNR devices with different widths, finding a perfect spin filtering effect reaching 100% over a wide bias range in both P and AP spin configurations for all systems. These predictions indicate that the edged phosphorus-doped ZGNRs have potential application in spintronic devices.

## Calculation Details

**Figure 1:**
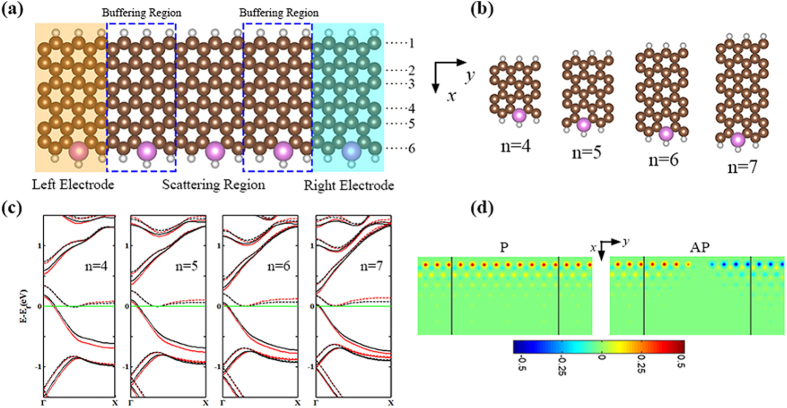
Schematic illustration of two-probe system and the spin charge density distribution. (**a**) Schematic illustration of a 6-ZGNR-P two-probe system. (**b**) The P-doped units cells of n-ZGNR (n = 4, 5, 6 and 7) along the transport direction 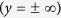. The brown, violet and the light gray balls represent carbon, phosphorus and hydrogen atoms, respectively. (**c**) Calculated spin-resolved band structures of the electrode units (with n = 4, 5, 6, and 7) by using the Nanodcal and VASP packages. The red solid and dashed curves are ↑-spin and ↓-spin channels of the Nanodcal package calculating results, and the black solid and dashed curves are ↑-spin and ↓-spin channels of the VASP calculating results, respectively. The Fermi level is placed at zero and is indicated by a green solid line. (**d**) The spin charge density distribution of 6-ZGNR-P system for the P and AP spin configurations under zero bias.

Figure 1a shows a single-edge phosphorus-doped n-ZGNR model device with width n = 6. The model device is divided into three regions: the left and right electrodes, and the scattering region. In our model, the carbon atoms at the edges are terminated by one hydrogen atom (sp^2^ type) in order to eliminate the dangling bonds. Here, the left and right electrodes are modeled by a semi-infinite replica of the doped unit (see Fig. 1b) extending along the transport direction *y* to 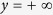 for right electrode and 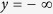 for left electrode. We assume that the doping is produced by substituting a single carbon (C) atom at the lower edge with a phosphorus (P) atom every three ZGNR unit cells. The right electrode is the same as the left one, and the scattering region contains three doped unit cells, where the left cell belongs to the left electrode, and the right cell belongs to the right electrode, which can screen the interaction between the electrodes and the center region, and serve as the buffering regions, as shown in Fig. 1a. We have checked that the charge density at left/right electrodes regions perfectly matches with that of the buffering region, which means that the design of the devices is reasonable. For convenience, we define the name of these systems as n-ZGNR-P (n = 4, 5, 6 and 7; P is phosphorus). A vacuum layer thicker than 15 Å is used to eliminate interactions between periodic images.

Optimization of all geometric structures is carried out within the framework of the density functional theory^31^ using the Vienna *ab initio* simulation package (VASP)^32^ with the projector augmented wave (PAW)^33^ method. The Perdew-Burke-Ernzernhof (PBE)^34^ generalized gradient approximation (GGA) is used as the exchange-correlation function. The plane-wave cutoff energy is set to 500eV and a 1 × 9 × 1 *k* mesh with the conjugate gradient algorithm was used. All the atomic positions are relaxed sufficiently until the energy and the force on each atom are less than 10^−5^ eV and 0.02 eV/Å, respectively.

After optimization, the subsequent spin-dependent transport properties of these systems were determined by using the NanoAcademic Device Calculator (Nanodcal)^35^,^36^,^37^ software package, which adopts the density functional theory (DFT) combined with the Keldysh nonequilibrium Green’s function formalism (NEGF). In the transport calculations, the local spin density approximation (LSDA)^38^,^39^,^40^ describes the exchange-correlation potential, and the valence electronic orbitals are expanded in a double-ζ plus polarization (DZP) basis set for all atoms. The cutoff energy for the real space grid is set to 190 Ry and 1 × 100 × 1 *k-*points mesh is employed in the Brillouin zone for electrodes. The spin-dependent current through the scattering region is obtained by the Landauer-Büttiker formula^41^:


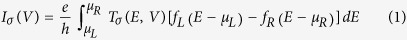


here, *h* and *e* are Planck’s constant and the charge of one electron, respectively. σ is ↑ (spin up) and ↓ (spin down), 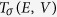 are spin-resolved transmission functions defined as:


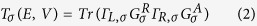


where 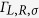 is the coupling matrix; 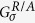 are the retarded and advanced Green’s functions of the scattering region, respectively. The transmission coefficient represents the probability that electrons at an energy *E* pass through the scattering region. 
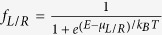
 are Fermi-Dirac distribution function of the left/right electrodes. 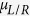 are electrochemical potentials of the left/right electrodes, and the difference between them is 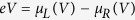 at a given bias *V*. For all practical purposes, the equilibrium Fermi level (*E*_*f*_) is zero in the two-probe system. The electrochemical potential of the right electrode has no shift for all bias, 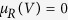. Just the Fermi energy of the left electrode shifts according to the bias. Therefore, the Fermi energy of the right electrode and scattering region is always zero. Thus, [-eV, 0] is the energy bias window (EBW), which means the energy region contributing to the current integral.

## Results and Discussion

To qualitatively verify the feasibility of our calculation results, we calculate the spin-resolved band structures of the electrode units (with n = 4, 5, 6, and 7) with the Nanodcal and VASP packages, as shown in Fig. 1c. Because the band structures greatly affect the transport properties discussed below. From Fig. 1c, we can find that the results calculated by the two methods agree well with each other. We confirm that the little difference only slightly affects the transmission. We also check the variation of band structures with the ribbon width and get consistent results. Therefore, our scheme of relaxing the structures using VASP and calculating transport properties using Nanodcal is reasonable.

Experimentally, the magnetization of the left and right electrodes can be aligned in parallel (P) or antiparallel (AP) spin configurations, which can be controlled by a sufficiently strong external magnetic field. Therefore, in our transport systems, the P and AP spin configurations are used to study the spin-polarized transport properties of all systems. Figure 1d shows the spin charge density distribution of 6-ZGNR-P system for the P and AP spin configurations under zero bias. From Fig. 1d, we can find that the spin polarization states are mainly localized at the undoped edge, giving rise to the magnetism at this edge, which is consistent with previous investigations^19^,^24^.

**Figure 2:**
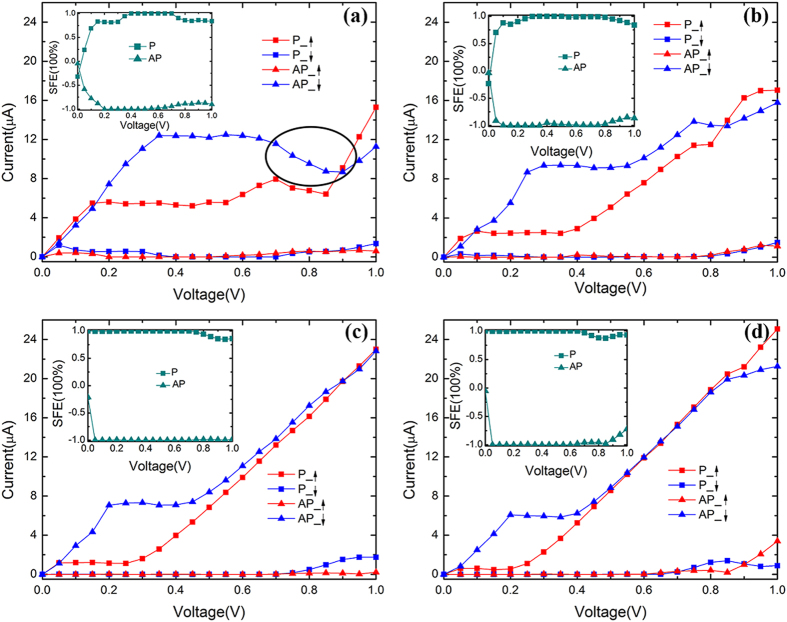
The spin-resolved current. The calculated spin-resolved currents for the P/AP spin configurations as a function of the bias (**a**) for 4-ZGNR-P, (**b**) for 5-ZGNR-P, (**c**) for 6-ZGNR-P and (**d**) for 7-ZGNR-P. The SFE as a function of bias is shown in the inset. The symbol ↑ and ↓ represents the spin up and down, respectively. The location of NDR for 4-ZGNR-P in (**a**) is marked with black oval line.

Figure 2 displays the spin-resolved *I*-*V* curves of the n-ZGNR-P (n = 4–7) systems for the P and AP spin configurations, respectively. As can be seen from the diagram, the distinct properties are as follows: (1) Perfect spin filtering effect: in the P and AP spin configurations, there are similar spin-resolved current trends with the increase of bias for all systems, especially for n = 5, 6 and 7. The current of ↑-spin electron for all of the P spin configurations is larger than that of ↓-spin one in whole bias range. For the P configuration, as bias increases, the current of ↑-spin electron changes obviously, while that of ↓-spin electron in a wide bias range is extremely small and almost zero. Spin-resolved current behaviors for AP and P spin configuration are just opposite. Thus, a perfect spin filtering can be expected for both P and AP spin configurations. In order to illustrate the spin filtering behaviors for all systems, the spin filtering efficiency (SFE) is defined as 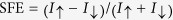. At zero bias, we obtain the SFE from the formula 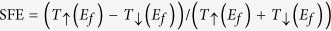, where 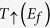 and 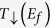 are the spin up and down transmission coefficients at the Fermi level, respectively. For the systems with different width, SFE as a function of bias is shown in the insets of Fig. 2. In 4-ZGNR-P system, the SFE for P spin configuration is almost 100% in the bias range from 0.4 V to 0.7 V and for the AP spin configuration the range is from 0.2 V to 0.6 V (insert of Fig. 2a). When n = 5, the bias range for SFE of P and AP spin configurations reaching 100% is more than that of the 4-ZGNR-P system, which are (0.3 V, 0.7 V) and (0.1 V, 0.8 V) (insert of Fig. 2b), respectively. As the width increases, the systems with n = 6 (insert of Fig. 2c) and 7 (insert of Fig. 2d) have a more perfect SFE with a wider range of bias. (2) As can be seen from the *I*-*V* curve in Fig. 2, all systems in both P and AP spin configurations have a current platform, namely, the current value within a certain bias range is almost the same. We take the 6-ZGNR-P system as an example to illustrate the *I*-*V* platform and linear relationship. In Fig. 2c, it is shown that the ↑-spin current for P spin configuration firstly increases with bias from 0 V to 0.05 V, but from 0.05 V to 0.25 V, the current is almost the same, that is, it is a platform. The value of platform current is about 1.2 μA and the current continues to increase after the platform. For the ↓-spin current in the AP spin configuration, the current platform appears in the bias region (0.25 V, 0.4 V) and the platform current is about 7 μA, which is larger than the current of P spin configuration at the same bias point. Similarly, the corresponding spin currents for P and AP spin configuration of the other systems also exhibit platforms. To sum up, from Fig. 2 we can analyze the ↓-spin platform current of AP spin configuration is larger than P spin configuration ↑-spin current with the same width for all systems. Secondly, as the width of the system increases, the platform current and platform widths for P and AP spin configurations have decreasing tendencies. In addition, in the 6-ZGNR-P and 7-ZGNR-P systems, both ↑ and ↓-spin currents of the P and AP spin configurations also exhibit a linear relationship as a function of bias when the bias exceeds that of the current platform region. Significantly, for the AP spin configuration, the 4-ZGNR-P system at the end of the ↓-spin current platform exhibits a negative differential effect (NDR) within the bias range (0.7 V, 0.9 V) (Fig. 2a).

**Figure 3:**
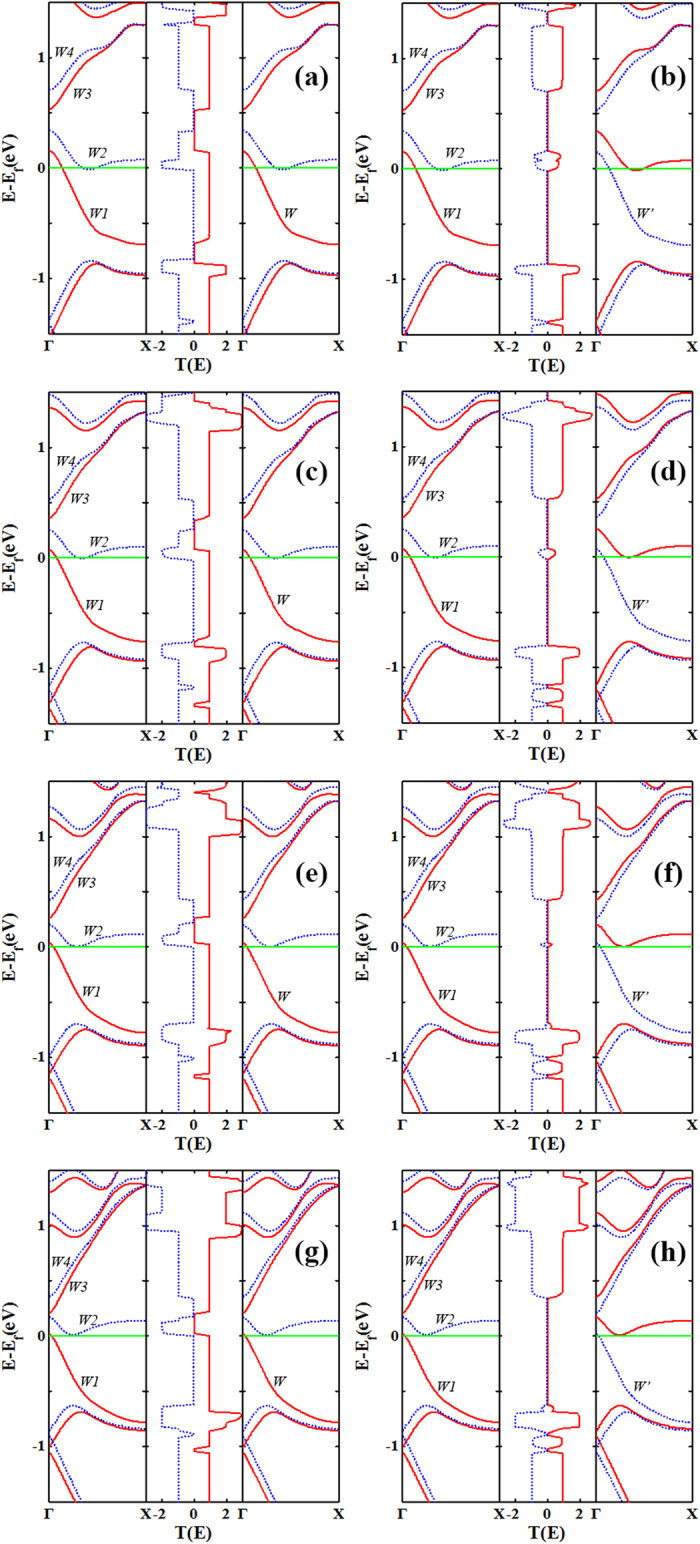
The spin-resolved band structures and the transmission spectrums. The spin-resolved band structures of left (left panels) and right (right panels) electrodes, and the transmission spectrums (middle panels) for the P (**a**,**c**,**e**,**g**) and AP (**b**,**d**,**f**,**h**) spin configurations under zero bias, from upper to lower panel corresponding to n-ZGNR-P (in turn, n = 4, 5, 6 and 7) systems. The red solid and blue dotted curves are for ↑-spin and ↓-spin channels, respectively. The Fermi level is placed at zero and is indicated by a green solid line.

To clarify the above interesting phenomena, we analyze the overlap of band structures of electrodes, and the transmission peak width and strength within the EBW for all considered systems at zero bias (Fig. 3). For convenience, we label the bands of left electrode through and above the Fermi level with the symbol *W1*, *W2, W3* and *W4*, and ↑-spin (P) and ↓-spin (AP) bands of the right electrode in the vicinity of the Fermi level with the symbol *W* and *W’*, respectively. From Fig. 3, for the P and AP configurations, we can find that the transmission spectra and the band structures of electrodes are quite similar in these systems. With the increase of n (n = 4, 5, 6 and 7), for the P configuration of all systems (Fig. 3a,c,e,g), the transmission peak of the ↑-spin channel has a tendency to shift downward near the Fermi level, while that of the ↓-spin channel changes very little, especially in a wide energy range there is no transmission spectrum below the Fermi level. In contrast, for the AP spin configuration (Fig. 3b,d,f,h), in a wide energy range, the ↑-spin and ↓-spin transmission peaks in the vicinity of the Fermi level are almost symmetrical, and gradually decrease with increasing n until the transmission peak disappears at n = 7 (Fig. 3h). These phenomena can be deduced from the band structures of the electrodes. From Fig. 3, we can find that the bands W1, W3, W and W’ are more dispersive, while the band W2 is much narrower. As an example, for the AP spin configuration of 6-ZGNR-P (Fig. 3f) system, since the band *W’* of right electrode occupies widely, when the positive bias is applied, the bands of the left electrode shift downward and *W’* keeps unchanged, thus *W’* will overlap with *W2*, *W4*, but no band of the right electrode matches to band *W1* within the EBW. This will cause the ↓-spin transmission coefficients within the EBW to be large and wide, but there is a large transmission coefficients gap in the ↑-spin channel. Thus a perfect spin filtering behavior is anticipated in the AP spin configuration for 6-ZGNR-P system. Similarly, for the P spin configuration of 6-ZGNR-P (Fig. 3e) system, the change of band structures due to n increasing has the following rules: The band *W1* shifts downward, and band *W2* shifts upward. The gap between band *W1* (*W2*) and *W3* (*W4*) gradually becomes smaller. But the band *W2* is much narrower, while the width of band *W3* and *W4* increases. As the width increases, for the ↓-spin channel of AP spin configuration, the platform current gradually becomes smaller due to the band *W2* becoming narrower, while the current platform gradually becomes narrower not only related to the narrowing of band *W2*, but also the narrowing of the gap between band *W2* and *W4*. In other words, the larger the width of nanoribbons, and the faster the overlapping of band *W4* with *W’* within the EBW at the smaller bias, then the narrower the platform width. And for the ↑-spin channel of P spin configuration, as the width increases, the platform current becomes smaller due to the reduction of the overlap of band *W1* with *W* induces the transmission coefficient area within the EBW to decrease. However, the platform width becoming narrower is associated with the reduction of the gap between band *W1* and *W3*. Regarding linear behavior of the *I*-*V* curve at different widths, as the width increases, the corresponding spin channels of 6-ZGNR-P and 7-ZGNR-P systems show a linear *I*-*V* curve. The narrowing of band *W2* is a reason for the size of the band gap, but the growing broadening of the bands *W3* and *W4* with n increasing is the primary reason. Moreover, because the left/right electrodes and the scattering region are the same material in these systems, the transport properties of these devices are not only similar but also they show a regular change with the increasing of device width.

**Figure 4:**
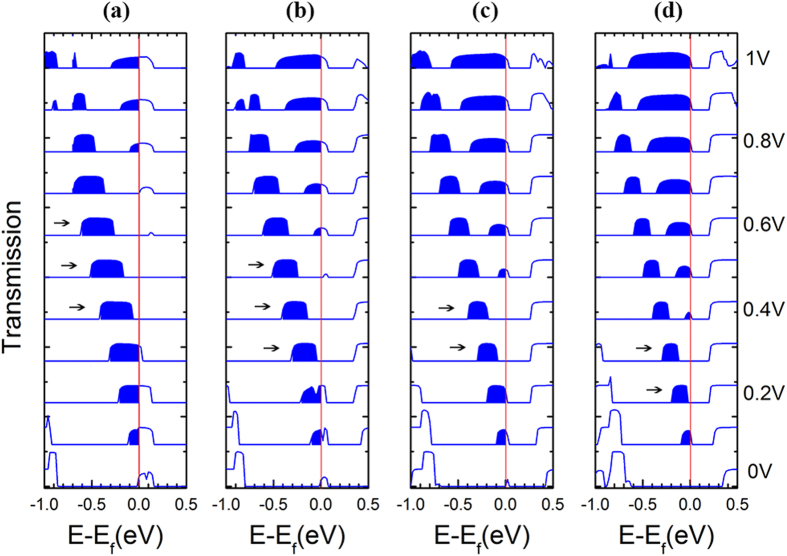
The transmission spectrums. The transmission spectrums for the AP configuration at the bias from 0 V to 1 V in steps of 0.1 V, (**a**) for 4-ZGNR-P, (**b**) for 5-ZGNR-P, (**c**) for 6-ZGNR-P, and (**d**) for 7-ZGNR-P. The blue area denotes the current integral energy region, and the integral region at the platform voltage range is marked by the black arrows. The Fermi level is placed at zero and is indicated by a green solid line.

In order to gain a better understanding of the NDR effect in the 4-ZGNR-P system, and the linear relationship between the current and bias in the 5-ZGNR-P, 6-ZGNR-P and 7-ZGNR-P systems, we take the AP configuration as an example, then plot the ↓-spin transmission spectra at the bias from 0 to 1 V in steps of 0.1 V for the all systems, as shown in Fig. 4. We can see from Fig. 4a, as the bias increases, the current integral area (the blue region) firstly increases, but at the bias range about (0.4 V, 0.6 V) (marked with the black arrows in Fig. 4a) remains the same, resulting in a current platform. However, when the bias is greater than 0.6 V, the integral region has decreasing tendency until bias reaching to 0.85 V, then the *I*-*V* valley appears, as a consequence, there is NDR behavior. By contrast, we take the 6-ZGNR-P as an example, as shown in Fig. 4c, when the bias is larger than 0.5 V, the transmission peak within the EBW near the Fermi level that is caused by the overlap of band *W4* and *W’* begins to gradually increase, while the other transmission peak of the current integral region that is caused by the overlap between band *W2* and *W’* changes little in a wide bias range, resulting in a linear increase of current. From Fig. 4b,c,d, we can also clearly see the similar mechanism that induces the platform effect and the *I*-*V* curve linear relationship in the 7-ZGNR-P system. In addition, we can also deduce from Fig. 4, as the system width increases, the integral region tends to decrease in the platform voltage range (marked with the black arrows in Fig. 4), that is to say, all platform currents for the AP spin configuration have a decreasing tendency.

**Figure 5:**
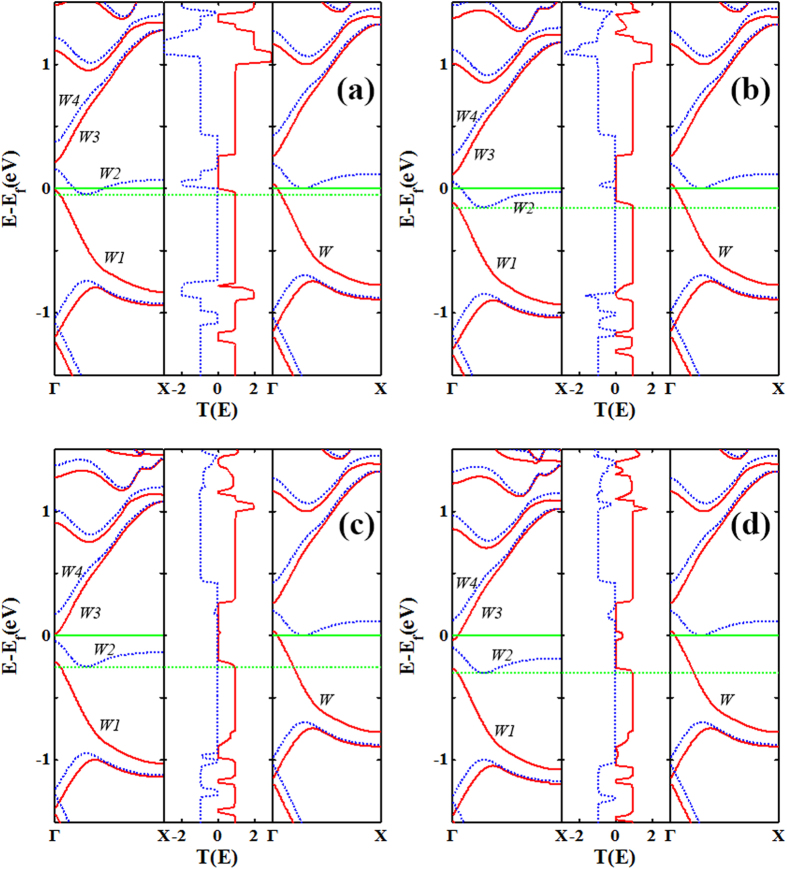
The spin-resolved band structures and the transmission spectrums. The spin-resolved band structures of left (left panels) and right (right panels) electrodes and the transmission spectrums (middle panels) for the P spin configuration in 6-ZGNR-P system at (**a**) 0.05 V, (**b**) 0.15 V, (**c**) 0.25 V, and (**d**) 0.3 V, respectively. The red solid and blue dotted curves are for ↑-spin and ↓-spin channels, respectively. The green dotted line denote the chemical potentials of the left electrode. The Fermi level is placed at zero and is indicated by a green solid line.

**Figure 6:**
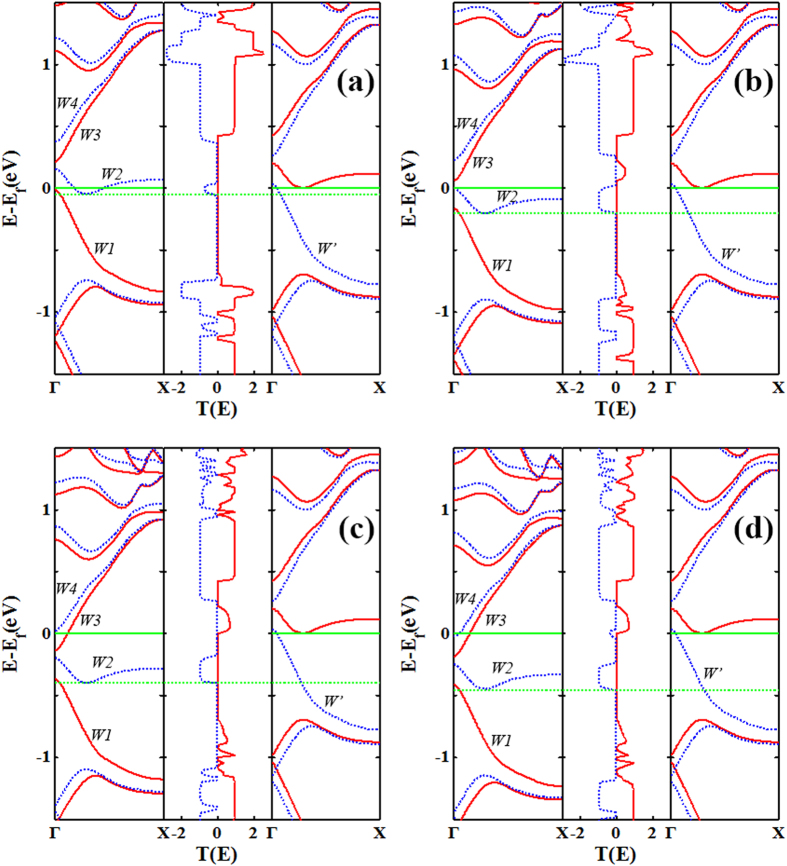
The spin-resolved band structures and the transmission spectrums. The spin-resolved band structures of left (left panels) and right (right panels) electrodes and the transmission spectrums (middle panels) for the AP spin configuration in 6-ZGNR-P system at (**a**) 0.05 V, (**b**) 0.2 V, (**c**) 0.4 V, and (**d**) 0.45 V, respectively. The red solid and blue dotted curves are for ↑-spin and ↓-spin channels, respectively. The green dotted line denote the chemical potentials of the left electrode. The Fermi level is placed at zero and is indicated by a green solid line.

To elucidate the physical mechanism for the peculiar properties of current performance, we take the 6-ZGNR-P transport system as an example. We plot the spin-resolved transmission spectra with the corresponding band structures of the left and right electrodes for the P spin configuration at bias 0.05 V, 0.15 V, 0.25 V and 0.3 V (Fig. 5), and that for the AP spin configuration at 0.05 V, 0.2 V, 0.4 V and 0.45 V (Fig. 6). For the P configuration, as shown in Fig. 5a, at 0.05 V, the band *W1* is overlapping with *W* within the EBW, causing the ↑-spin current be non-zero. Similarly, as bias 0.15 V (Fig. 5b) and 0.25 V (Fig. 5c) is applied, the transmission coefficient area for the ↑-spin channel within the EBW is almost the same for these three biases, therefore, the value of the current is the same and the *I*-*V* curve platform appears. When the voltage increases to 0.3 V (Fig. 5d), the ↑-spin channel transmission coefficient area within the EBW consists of two parts, as well as the part that is generated by the overlap between the band *W1* and *W*, a new transmission peak produced by the overlapping of band *W3* and *W* appears and contributes to the integral area. As a result, comparing bias at 0.3 V with 0.25 V, the ↑-spin transmission channel augments, thus, the ↑-spin current starts to increase. On the other hand, for the ↓-spin channel, since there is no band of right electrode matching to band *W2* and *W4* of left electrode within the EBW, so there is no band overlap in a wide bias range, then there are virtually no ↓-spin electrons through the scattering region, making the ↓-spin current zero, so that a perfect spin filtering is obtained in the P spin configuration. For the AP spin configuration, as shown in Fig. 6, at 0.05 V (Fig. 6a), the band *W2* and *W’* have a overlap within the EBW, resulting in a little peak in the ↓-spin channel transmission spectra. As the positive bias increases, the bands of the left electrode continue to shift downward, while the bands of the right electrode don’t move in our calculations. As a result, in the progress of band *W2* coming into the EBW, the ↓-spin current will continue to increase. At 0.2 V (Fig. 6b), the band *W2* has completely moved into the EBW. Because there is a gap of about 0.25 eV between the band *W2* and *W4*, therefore, the ↓-spin current will remain the same until the overlap between the band *W4* and *W* (at 0.45 V, as shown in Fig. 6d) is within the EBW. That is to say, the current platform occurs in the bias range (0.2 V, 0.4 V). However, for the ↑-spin channel, within the EBW, since there is no corresponding overlap of the bands of the left/right electrodes in the low bias range, thus, there are no ↑-spin electrons passing through the scattering region. Furthermore, regarding the fact that the magnitude of the platform current for the ↓-spin channel of the AP spin configuration is larger than the ↑-spin channel of the P configuration, that is mainly because the overlap between the band *W2* and *W’* is stronger than that of the band *W1* and *W* within the EBW at the corresponding platform bias. The intensity of the ↓-spin transmission peak in the AP spin configuration is greater than the ↑-spin transmission peak in the P spin configuration within the EBW. Similarly, we can also explain the current behavior of 4-ZGNR-P, 5-ZGNR-P and 7-ZGNR-P systems.

## Conclusions

In conclusion, we have investigated the spin-dependent electronic transport properties of n-ZGNR-P (n = 4, 5, 6 and 7) systems by using NEGF-DFT approach and found that a perfect spin filtering effect reaches 100% in a wide bias range in both P and AP configurations for all systems, especially for 6-ZGNR-P system. In addition, our calculations also show a NDR behavior of 4-ZGNR-P system in AP spin configuration and a current platform in all systems. In particular, monotonic increasing of the current with bias in the 6/7-ZGNR-P systems can be well observed. Furthermore, the variation of transport properties with the increasing device width is well presented and explained. The physical mechanism for these peculiar properties is that the change of band structures due to nanoribbon width and the shift of them according to the biases generate different overlap of band structures between left and right electrodes, leading to different transmission peak width and strength within the EBW for all considered systems, then the transport properties of these devices are not only similar but also they show a regular change with the increasing of device width.

## Additional Information

**How to cite this article**: Zou, F. *et al.* Perfect spin filtering effect and negative differential behavior in phosphorus-doped zigzag graphene nanoribbons. *Sci. Rep.* **5**, 15966; doi: 10.1038/srep15966 (2015).
